# Stepwise Tfh cell differentiation revisited: new advances and long-standing questions

**DOI:** 10.12703/r/10-3

**Published:** 2021-01-15

**Authors:** Andrew R Schroeder, Fangming Zhu, Hui Hu

**Affiliations:** 1 Department of Microbiology, University of Alabama at Birmingham, Birmingham, AL 35294, USA

**Keywords:** Tfh, stepwise differentiation, germinal center, TCR, metabolism, CXCR5, transcription factor, CD4 memory

## Abstract

T follicular helper (Tfh) cells play an essential role in germinal center formation and the generation of high-affinity antibodies. Studies have proposed that Tfh cell differentiation is a multi-step process. However, it is still not fully understood how a subset of activated CD4^+^ T cells begin to express CXCR5 during the early stage of the response and, shortly after, how some CXCR5^+^ precursor Tfh (pre-Tfh) cells enter B cell follicles and differentiate further into germinal center Tfh (GC-Tfh) cells while others have a different fate. In this mini-review, we summarize the recent advances surrounding these two aspects of Tfh cell differentiation and discuss related long-standing questions, including Tfh memory.

## Introduction

Upon activation, naïve CD4^+^ T cells have the potential to differentiate into distinct subsets of effector T helper cells with varied functions^1^. T follicular helper (Tfh) cells are a unique CD4^+^ T cell subset that provides help to B cells and is essential for germinal center (GC) formation^2–5^. The differentiation and function of Tfh cells have been shown to determine the kinetics and magnitude of GC B cell responses and the generation of high-affinity antibodies^4,5^.

Tfh cell differentiation has been proposed to be a multi-stage process^4,6–8^. During the early stage of CD4^+^ T cell responses, antigen presentation by dendritic cells (DCs) activates naïve CD4^+^ T cells and initiates Tfh cell differentiation, including the induction of CXCR5 expression^9^. Besides T cell receptor (TCR) signaling, co-stimulation and the local cytokine milieu are critical in Tfh fate determination^7,10–16^. After the DC priming stage, further Tfh cell differentiation requires interactions with B cells^7,10,17^. Intriguingly, it seems that not all of the activated CXCR5^+^CD4^+^ T cells will enter B cell follicles and become germinal center Tfh (GC-Tfh) cells, as some activated CXCR5^+^CD4^+^ T cells have been shown to develop into memory CD4^+^ T cells that are multi-potent^18^.

In the past decade, great progress has been made in our understanding of Tfh cell differentiation and function. Nonetheless, the molecular underpinning of CXCR5 induction and the signals that govern the divergence of Tfh versus non-Tfh effector cells in the early stage of CD4^+^ T cell responses are still not fully understood. In addition, with improved methods and more detailed mechanistic studies being carried out, it has become clear that the broadly defined “CXCR5^+^ Tfh” phenotype is too simplistic and may lead to confusion—especially regarding Tfh cell fate determination and Tfh memory. Here, we discuss recent advances in Tfh cell differentiation and highlight some important unanswered questions.

## DC priming for initial Tfh cell differentiation

DCs are superior antigen-presenting cells (APCs) for naïve CD4^+^ T cell activation and differentiation^19^. Early studies have shown that during the initiation of the CD4^+^ T cell response, DCs were sufficient to prime naïve CD4^+^ T cells and generate CXCR5^+^ Tfh cells^9^; however, the further differentiation into GC-Tfh cells still required B cell interactions^7,9,10,17^. Based on surface markers, location, and functional differences, several subsets of DCs have been identified^20,21^; thus, determining whether the DC subsets have different capabilities to generate Tfh responses has been an active area of research.

The two major populations of murine DCs are conventional DCs (cDCs) and plasmacytoid DCs (pDCs)^20–22^. The zinc finger transcription factor Zbtb46 (zDC) is selectively expressed by cDCs but not by pDCs or other myeloid cells^23,24^. The cDC population has been targeted by the introduction of the human diphtheria toxin receptor (DTR) into the *zDC* gene to create zDC-DTR mice, such that zDC-expressing cDCs would be subject to diphtheria toxin (DT) treatment-mediated deletion^23^. In these mice, DT treatment led to the near complete block of CD4^+^ and CD8^+^ T cell activation and proliferation^23^. cDCs can be further classified into cDC1s and cDC2s, which can be resident or migratory and have identifying surface markers that may depend on location^20–22^. Previous studies have shown that in the absence of migratory cDC2s, resident cDC1s in lymph nodes were capable of inducing Tfh and humoral responses^25,26^. Recently, using Batf3 knockout (KO) mice (lacking cDC1s, with preserved cDC2s) and Dock8 KO mice (preserved cDC1s but lacking cDC2s), Krishnaswamy *et al*. showed that the migratory cDC2s, but not cDC1s, uniquely induced Tfh cell responses at the T-B border^27^. This finding corroborates an earlier independent study on EBI2 function in CD4^+^ T cells^28^. EBI2, encoded by *Gpr183*, is an orphan G protein-coupled receptor that was first shown to have a critical function in the localization of activated B cells to the outer follicle and the induction of early plasmablast and antibody responses^29,30^. Following this, Li *et al*. found that the Tfh cell differentiation of EBI2-deficient CD4^+^ T cells was compromised in the early stage of the response in a B cell-independent manner^28^. In EBI2-sufficient CD4^+^ T cells, most of the activated cells were co-localized with the activated DCIR2^+^ cDC2s in the outer T zone within 12 hours to 2 days after antigen challenge^28^. In the absence of EBI2, activated CD4^+^ T cells were scattered and only some were in the vicinity of DCIR2^+^ cDC2s^28^. The study went further to reveal a novel mechanism underlying this important T–DC interaction at the inter-follicular region in Tfh cell development: the activated CD4^+^DCIR2^+^ cDC2s expressed and released soluble CD25 (IL-2Rα) to absorb IL-2^28^, thereby significantly reducing IL-2-mediated suppression of Tfh cell differentiation, as previously reported^14,16^.

Although cDC2s seem to play a larger role in generating Tfh responses, studies have also shown that, under certain circumstances, DCs may not be absolutely required for Tfh cell differentiation. In the study of EBI2 on T–DC interaction in Tfh cell differentiation, the deletion of cDCs in the zDC-DTR mice completely abolished Tfh cell differentiation in response to SRBC-OVA immunization^28^. Yet using the same zDC-DTR mouse model, Arroyo *et al*. found that in CD4^+^ T cell responses to *Plasmodium*, a blood-borne parasite, the CXCR5^+^ Tfh cells generated in DT-treated zDC-DTR mice were at levels similar to those of wild-type mice^31^. Meanwhile, in the B-MHC II model in which MHC class II was expressed only on CD19^+^ B cells^31^, CXCR5^+^ Tfh cells were still generated, suggesting that B cells are sufficient to generate Tfh cells in *Plasmodium* infection^31^. Additionally, a 2015 study showed that when very high doses of antigens were used, the defective Tfh cell differentiation in the absence of DCs could be overcome as well^32^.

In addition to antigen delivery route and dose, antigen size can apparently have a selective impact on Tfh cell differentiation^33^. Another 2015 study showed that compared with an antigen size of 40 or 1000 nm, the antigen size of about 200 nm induced higher Tfh cell responses and antibody responses despite a similar extent of total OT-II T cell responses^33^. Although the exact mechanism is still not clear, the study provided evidence to suggest that increasing particle size (to a certain level) leads to sustained antigen presentation by DCs and maintained T–DC interactions, resulting in enhanced Tfh cell differentiation^33^.

Although most studies on DCs have focused on their function to promote Tfh cell differentiation, a unique DC subset has been found to play a preferential negative role in regulating humoral responses. Kumamoto *et al*. showed that the CD301b^+^ DCs of migratory CD11b^+^ dermal DCs expressed high levels of the PD-1 ligands 1 and 2 (PD-L1 and PD-L2), and the depletion of this DC subset resulted in enhanced Tfh, GC B cell, and antibody responses to protein antigens even in the absence of adjuvants^34^.

Thus far, depending on the infectious agent, site and route of immunization, type of adjuvant, and antigen size and amount, the collected studies of various model systems suggest that almost all types of cDCs have the capacity to stimulate and generate CXCR5^+^ Tfh cell responses, but the cDC2s appear to play a more dominant role to inhaled antigens^20,21,27^. With regard to DC priming during the initial stage of Tfh cell differentiation, one of the remaining long-standing questions goes back to the original issue of the divergence of naïve CD4^+^ T cells upon activation: what signals drive activated CD4^+^ T cells to become CXCR5^–^ versus CXCR5^+^ cells? At present, the molecular mechanism underlying the induction of CXCR5 expression is still incompletely understood.

## CXCR5 induction

Tfh cells were initially identified and defined as CXCR5^+^CD4^+^ T cells in the B cell follicles^2,3^. Functionally, when activated CD4^+^ T cells downregulate CCR7 expression, the upregulation of CXCR5 has been shown to be involved in T cell migration into the B cell follicles^35,36^.

Studies have shown that IL-21, inducible co-stimulator (ICOS), Bcl6, and likely NFAT are important for CXCR5 expression^7,10,37^, yet how to consistently induce CXCR5 expression in CD4^+^ T cells *in vitro* has remained a challenge^38^. It was remarkable when the overexpression of helix-loop-helix (HLH) transcription factor Ascl2 alone was found to be sufficient to induce CXCR5 expression *in vitro* and that study went further to suggest that Ascl2 induction initiates Tfh cell programming^39^. Later, in another independent study on Id families and HLH E proteins, the transcription factors E12 and E47 (encoded by *Tcf3*) were also shown to drive CXCR5 expression *in vitro*^40^, suggesting that HLH proteins play critical roles in regulating CXCR5 expression. Surprisingly, in the lymphocytic choriomeningitis virus (LCMV) infection model, whereas *Tcf3* was induced in activated CD4^+^ T cells in the early days of the response, the expression of *Ascl2* was not detected when CXCR5^+^ Tfh cells already started to emerge^40^. That study on Id and E proteins went further to reveal that Id2 suppressed E12/E47 (E2A)-mediated CXCR5 upregulation *in vitro*, although Id2 did not seem to be able to inhibit Ascl2-medicated CXCR5 upregulation^40^, suggesting that the Ascl2 expression in the later stage of Tfh cell differentiation may still be important. Interestingly, Id2 was shown to be a target of Bcl6, and Bcl6 directly repressed Id2 expression in CD4^+^ T cells^40^, providing evidence of one mechanism by which Bcl6 regulates CXCR5 expression.

In the immune system, transcription factor Bach2 was first discovered as a key player in antibody class switching^41^. Later, in T cells, it was found that Bach2 is involved in CD8^+^ T cell memory and regulatory T (Treg) cell homeostasis^42–45^ and is important for restraining Th1, Th2, and Th17 cell differentiation as well^42,46^. Studies have also shown that Bach2 directly targets and negatively regulates Blimp1^44,47,48^. Thus, it was a total surprise that the deletion of Bach2 in CD4^+^ T cells led to preferential Tfh cell differentiation^49–51^ despite increased Blimp1 expression and effector functions^50,51^. Mechanistically, Bach2 was found to be a critical early regulator of CXCR5 expression, such that enhanced *Cxcr5* expression in the absence of Bach2 occurred even before the induction of *Ascl2* expression^50^. The transcriptome analysis of naïve and activated CD4^+^ T cells showed that *Tcf3* and *Id2* expression levels were not significantly affected by the absence of Bach2 on day 3 in a protein immunization model^50^, suggesting that Bach2-mediated control of CXCR5 expression is independent of and most likely prior to E2A- or Id2-mediated regulation.

In addition to a Blimp1- and E2A-dependent regulatory element in the CXCR5 intron and a Blimp1-dependent regulatory element upstream of the promoter^52^, a new regulatory element 36 kb upstream of the murine CXCR5 locus which suppresses *Cxcr5* promoter activity in a Bach2-dependent manner in reporter assays *in vitro* was identified^50^. Nevertheless, despite the reporter activities, neither wild-type nor Bach2-deficient CD4^+^ T cells upregulated CXCR5 expression on the cell surface *in vitro*^50^, suggesting that other layers are involved in the regulation of CXCR5 expression *in vivo*.

Although some transcription factors have been identified to promote or suppress CXCR5 expression, the upstream signaling events that control these transcription factors and lead to the induction of CXCR5 have not been well defined. In addition, whereas CXCR5 helps to define Tfh cells, some studies have shown that it may not be absolutely necessary for Tfh cell localization into the GC. An adoptive transfer study using T cell-deficient recipient mice^53^ and a newer study using an influenza infection model in *Cxcr5*^f/f^ CD4-Cre mice^54^ showed that it was possible for CXCR5-deficient CD4^+^ T cells to enter B cell follicles and form GC-Tfh cells, suggesting that some other signals allow for the GC localization of CXCR5-deficient cells. Nevertheless, earlier studies demonstrating that CXCR5-deficient CD4^+^ T cells failed to enter the B cell follicle when adoptively transferred into CXCR5-sufficient, lympho-replete recipients^35,36^ indicate that CXCR5 upregulation is important in a competitive environment. More work is warranted to understand the regulation and function of CXCR5 in Tfh cell differentiation.

## TCR affinity and signal strength in Tfh cell differentiation

Using 5C.C7 (high-affinity) and 2B4 (low-affinity) PCC-specific TCR transgenic T cells in both co-transfer and independent adoptive transfer experiments, an early study by Fazilleau *et al*. found that significantly more 5C.C7 CD4^+^ T cells than 2B4 CD4^+^ T cells developed into CXCR5^+^ Tfh cells, suggesting that a high-affinity TCR may lead to preferential Tfh differentiation of CD4^+^ T cells^55^.

The link between TCR affinity and Tfh cell differentiation was further studied by tracking the progeny of adoptively transferred single naïve CD4^+^ T cells^56^. Tubo *et al*. showed that naïve CD4^+^ T cells specific to a certain peptide:MHC II underwent distinct patterns of CD4^+^ T helper cell differentiation based on unique TCRs^56^. As the productive TCR signal is decided not only by TCR affinity but also by the aggregate half-life of TCR:MHC interaction, the study showed that when the TCR-peptide:MHC II dwell time continued to increase, the Th1 cell differentiation would peak and decrease whereas the Tfh cell differentiation would increase and reach a plateau^56^. Additionally, increased doses of antigens—leading to stronger TCR signaling—seemed to favor Tfh and GC-Tfh cell development^56,57^.

Studies in viral infection models have shown that the IL-2/STAT5 pathway suppresses Tfh cell differentiation^14,16^. Using the IL-2-GFP reporter mice, both *in vitro* and in the ActA-deficient *Listeria monocytogenes* infection model *in vivo*, a recent study reported that within 24 hours, newly activated CD4^+^ T cells already split into two populations: IL-2^+^ cells enriched for *Bcl6* mRNA expression and IL-2^–^ T cells expressing higher mRNA levels of *Prdm1*, *S1pr1*, and *Klf2*^58^. Then, using the IL-2-Thy1.1 reporter mice, the study showed that Thy1.1 (IL-2) expression was restricted to CXCR5^+^CD4^+^ T cells and that treatment of anti-Thy1.1 antibodies—leading to the depletion of IL-2-producing cells—abolished Tfh cell differentiation, suggesting that CXCR5^+^ Tfh cells are derived from IL-2 producers^58^. The study further showed that IL-2 production and Tfh cell differentiation correlated with TCR signal strength and that higher TCR signaling favored Tfh cell development^58^.

Interestingly, it has also been reported that low-affinity antigen led to unimpaired Tfh cell differentiation^59^ and that high-affinity TCR favored Th1 over Tfh differentiation^60^. However, it should be noted that because TCR affinity positively correlated with cell expansion, higher-affinity TCRs led to more responding cells, including the Tfh population^60^. That study found that high-affinity TCR led to sustained CD25 expression and induced higher levels of TCR-regulated genes—such as *Eef1e1*—that would promote non-Tfh effector cell differentiation in a T cell-intrinsic manner^60^.

How TCR signal strength translates into downstream transcriptional regulation that will influence the subsequent T cell differentiation has been a long-standing question. Recently, using the 5C.C7 TCR transgenic T cells and peptides with different binding affinities, Krishnamoorthy *et al*. found that the amounts of transcription factor IRF4, an immediate-early target gene of TCR signaling^61^, were increased proportionately to TCR signal strength^62^. Whereas low levels of IRF4 were critical for Tfh cell differentiation, the increased TCR signaling raised IRF4 to high levels, which in turn constrained the Tfh cell differentiation and favored the development of non-Tfh effector development^62^. The TCR signal strength/IRF4 level-mediated control of T helper cell fate was attributed to the divergent DNA motifs that the low and high levels of IRF4 target, and greater IRF4 levels allowed binding to low-affinity binding sites that were enriched in non-Tfh effector genes, and the process was independent of IL-2 signaling^62^.

The above studies have investigated the role of TCR affinity/signaling in Tfh differentiation—in particular, the Tfh versus non-Tfh effector fate decision—yet it is still controversial when CXCR5^–^ non-Tfh versus CXCR5^+^ Tfh divergence occurs upon naïve CD4^+^ T cell activation. Studies using an LCMV model showed that this could occur as early as the second or third cell division by day 2 or 3 after infection^7,63,64^. Meanwhile, in a recent study using single-cell RNA sequencing to map the development of CD4^+^ T helper cell differentiation in a *Plasmodium* infection model, the computational analysis of a temporal mixture of Gaussian processes model (GPfates) of non-Tfh and Tfh fate bifurcation suggested that Th1 and Tfh cells start to diverge at the time point after the initial burst of cell proliferation, associated with an upregulation of aerobic glycolysis and accelerated cell cycling^65^. This is consistent with the findings showing that CXCR5 expression was induced in activated CD4^+^ T cells that had proliferated most^66,67^. It is worth pointing out that early studies using protein immunization models have reported that there is an early but transient upregulation of CXCR5 and Bcl6 proteins in activated CD4^+^ T cells^66,68^. It is possible/plausible that the timing of the CXCR5^–^ non-Tfh versus CXCR5^+^ Tfh divergence varies in response to different antigens/infections. Nevertheless, the sustained increase of Bcl6 expression eventually marks the differentiation of Tfh cells, consistent with the role of Bcl6 as the central regulator of Tfh cell differentiation^69–71^. The regulation of Bcl6 expression in activated CD4^+^ T cells, however, is still under intense investigation.

## New factors that regulate Bcl6 and Tfh differentiation

Many transcription factors have been discovered to play important roles in Tfh cell development^10,15,16,37,39,62,69,72–84^. Adding to this, a number of new factors to regulate Bcl6 expression and function as well as Tfh cell differentiation have recently been identified.

## TCF1/LEF-1

TCF1 (encoded by *Tcf7*) and LEF1 (encoded by *Lef1*) are transcription factors containing a conserved high-mobility-group DNA-binding domain and have been shown to be important for early T cell development and memory CD8^+^ T cells^85–89^. TCF1 has also been reported to be involved in Th2 cell differentiation and IL-17A production^90,91^. In naïve CD4^+^ T cells, both TCF1 and LEF1 are expressed at high levels^92^. Three 2015 studies showed that the deletion of TCF1 or LEF1 (or both) resulted in impaired Tfh cell differentiation^63,92,93^. In the LCMV infection model, within 3 days of infection, it was found that the activated CD4^+^ T cells had already divided into TCF1^+^Blimp1^–/lo^ versus TCF1^–/lo^Blimp1^+ ^populations and that the TCF1^+^ population later differentiated into Tfh cells^63^. In addition, there seemed to be an antagonistic network between the IL-2/Blimp1 axis and TCF1: IL-2 signaling and Blimp1 suppressed TCF1 expression and favored Th1 effector cell differentiation, while TCF1 negatively regulated CD25 and Blimp1 expression, promoting Tfh cell differentiation^63^. TCF1 has also been shown to negatively modulate the expression levels of Th1 regulators T-bet and Id2 in a Blimp1-dependent manner^94^. In addition, TCF1 has multiple isoforms due to differential promoter usage and alternative splicing^95^. It was discovered that TCF1 long isoforms (p45 and p42) containing an N-terminal β-catenin-binding domain were required for Tfh cell differentiation and memory Tfh cells^95^ and that TCF1 and Blimp1 were mutual direct targets^63,94^. In the LCMV model, TCF1 was also found to bind a large number of Tfh genes, including *Il6st* and (in particular) *Bcl6*^92,93^. Loss of TCF1 also resulted in reduced cell proliferation and increased apoptosis^92,93^.

An intriguing point of the TCF1 function is that it seems the role of TCF1 in Tfh cell differentiation may be specific to immune responses against viral infections^63,92,93^. It was mentioned that in a protein immunization model, the Tfh cell development was TCF1-independent^63^. In agreement with this observation, almost no CD25 expression was detected in activated CD4^+^ T cells in NP-OVA antigen challenge *in vivo* (F.M. Zhu and H. Hu, unpublished data). It is conceivable that the regulatory mechanisms of Tfh cell differentiation vary in cells responding to different types of infections/challenges and this possibility warrants more investigation.

## Ezh2 and Nsd2

The histone methyltransferase (HMT) Ezh2 catalyzes H3K27 trimethylation and has been shown to play important roles in Th1, Th2, and Treg cells via its HMT activity^96–100^. Recently, a study by Li *et al*. unveiled novel and unexpected mechanisms by which Ezh2 regulates Tfh cell differentiation^101^. Ezh2-deficient CD4^+^ T cells were activated at a frequency similar to that of wild-type CD4^+^ T cells^101^. However, the deletion of Ezh2 resulted in severely defective Tfh cell development^101^. Ezh2 interacts with DNA only in coordination with binding partners; for this reason, the authors combined transcription factor and histone ChIP-seq (chromatin immunoprecipitation followed by sequencing) analysis and gene set enrichment analysis and found that almost half of the Ezh2 binding peaks overlapped with TCF1 peaks^101^. These two transcription factors activated a common subset of target genes in the Tfh cell program, with 70% of Ezh2/TCF1 co-occupied regions located in the gene promoters^101^. More detailed analyses showed that Ezh2, in its Ser21 phosphorylated form, cooperated with TCF1 and directly regulated Bcl6 expression^101^. In Tfh cells, surprisingly, Ezh2 was found to be associated with more H3K27ac and active gene transcription than H3K27me3 and gene repression^101^. Yet Ezh2 HMT activity is still important, as Ezh2 repressed p19Arf, an alternative splicing product of *Cdkn2a*, to promote Tfh cell survival^101^. Another interesting discovery was that p19Arf was found to directly interact with and antagonize Bcl6 function^101^. Thus, in Tfh cell differentiation, Ezh2 not only helps increase the Bcl6 expression but also prevents p19Arf from antagonizing the Bcl6 function, revealing a multifaceted role for Ezh2 in Tfh differentiation^101^.

In a parallel study, Chen *et al*. also discovered and emphasized that Ezh2 is important for the early commitment of Tfh cells^64^. The study combined ATAC-seq (assay for transposase-accessible chromatin using sequencing) and histone ChIP-seq to demonstrate that in the LCMV model, in which Th1 and Tfh cell differentiation programs started to diverge as early as day 2 after infection, Ezh2 was critical for the remodeling of chromatin accessibility of a cluster of Tfh-associated genes, including the *Bcl6* locus in particular^64^.

Another HMT, Nsd2, has also been reported to play an important role in Tfh cell differentiation. Induced by CD28 stimulation, the H3K36me2 methyltransferase Nsd2 has been shown to be required for increased Bcl6 expression as early as the first cell division^102^. The increased Nsd2 expression after T cell activation was further sustained by ICOS signaling and this was important for the maintenance of Tfh cells^102^.

## Tox proteins

The HMG box transcription factor Tox is involved in CD4^+^ T cell development in the thymus, natural killer cell differentiation, and lymph node organogenesis^103^. Recently, this transcription factor received a lot of attention after its essential role in exhausted CD8^+^ T cell programming was revealed^104–108^. As a key molecular regulator of exhausted CD8^+^ T cell development, Tox was shown to recruit diverse chromatin remodeling factors to modulate chromatin accessibility and epigenetic changes, thereby regulating a network of transcription factors and their targets^105,108^. On the other hand, in CD4^+^ T cells *in vitro*, under Tfh-like conditions, a recent study showed that Tox2 proteins regulated the Tfh-associated transcriptional program by promoting chromatin remodeling and cooperative function with IL-6 signaling and the Tfh transcription factors *Bcl6*, *Ascl2*, and *Batf*^109^. That study further revealed that Bcl6 directly regulated Tox2 and that Tox2 inhibited Th1, Th2, and Th17 cell differentiation^109^. Interestingly, the study also showed that Tox and Tox2 shared many overlapping functions in Tfh cell differentiation and that the two transcription factors synergized in regulating T cell localization in GCs^109^.

## Thpok

Studies have shown that, during thymocyte development, Thpok is a CD4^+^ T cell lineage-specific transcription factor that promotes CD4^+^ T cell differentiation^110,111^. In an activation-induced deletion model, a recent study showed that Thpok was not required for CD4^+^ T cell expansion but was critical for Tfh cell differentiation and the subsequent GC B cell responses^112^. That study showed that Thpok acted upstream of Bcl6 and directly regulated Bcl6 expression, yet Bcl6 overexpression alone was not sufficient to rescue the defective Tfh cell development in the absence of Thpok^112^. A more detailed analysis showed that Thpok was responsible for establishing the Tfh cell transcriptome using a mechanism independent of the interference from Blimp1 or Runx3, controlling many genes important for Tfh function which are not regulated by Bcl6 (for example, *Cd40lg*)^112^. Finally, the rescue experiments revealed that Maf, together with Bcl6, helped restore Tfh cell differentiation^112^.

## Extracellular matrix protein 1

Extracellular matrix protein 1 (ECM1) is a secreted protein and its mutations can lead to the genetic disorder lipoid proteinosis^113^. In CD4^+^ T cells, ECM1 has been shown to regulate Th2 cell migration and Th17 cell differentiation^114,115^. Recently, He *et al*. found that *Ecm1* was expressed at much higher levels in Tfh cells than in non-Tfh cells and the cytokines IL-6 and IL-21 enhanced ECM1 expression in activated CD4^+^ T cells at both mRNA and protein levels^116^. The binding of STAT3 to the promoter and the first intron region of the *Ecm1* locus suggested that *Ecm1* is a direct target of STAT3^116^. The study also nicely demonstrated that ECM1 promoted Tfh cell differentiation in an autocrine manner^116^. Mechanistically, ECM1 functioned to preserve Bcl6 expression by antagonizing the IL-2–STAT5 signaling pathway^116^.

## Prkd2

Prkd2 is one of the three serine/threonine protein kinase D family isoforms that have been indicated to regulate cytokine production in activated T cells^117^. In a forward genetic screen, a missense mutation of Prkd2 and subsequently the generation of mice carrying this mutation or deficient in Prkd2 were all found to exhibit excessive antibody responses^118^. More in-depth analyses showed that the excessive GC B cell development and the increased serum IgE, IgG1, and IgA levels in Prkd2-deficient mice were Tfh-dependent^118^. Mechanistically, it was found that Prkd2 directly bound to Bcl6 and induced phosphorylation of Bcl6, which restricted Bcl6 nuclear translocation in CD4^+^ T cells^118^. The loss of Prkd2 resulted in increased Bcl6 amounts in nuclear fractions, thereby enhancing Tfh cell development. Interestingly, Bcl6 was shown to downregulate Prkd2 expression in CD4^+^ T cells^118^, forming a mutual inhibitory-positive feedback system that facilitates Tfh cell differentiation.

Despite the new advances in our understanding of the transcriptional program controlling Tfh cell differentiation—including the new players upstream of Bcl6—the question of Tfh fate determination still appears to be complicated. In particular, it seems that not all of the CXCR5^+^ precursor Tfh (pre-Tfh) will enter B cell follicles and become GC-Tfh cells^18,31^. In this regard, the question remains as to which CXCR5^+^Bcl6^+^CD4^+^ pre-Tfh cells will become GC-Tfh cells and which ones will have different fates.

## Tfh cell migration and position

For CXCR5^+^ pre-Tfh cells to enter B cell follicles and become GC-Tfh cells, cell migration is a crucial part of the process. Early studies have shown that SAP-mediated regulation of T-B interactions and ICOS-ICOSL-mediated cell motility are necessary for CXCR5^+^ pre-Tfh cells to enter B cell follicles and help form GCs^7,10,119,120^. ICOS stimulates PI3K signaling, and using a novel mouse model with a gain-of-function point mutation of the PI3K subunit p110δ (PI3Kδ), Preite *et al*. found that the mutant PI3Kδ was able to induce enhanced Tfh cell differentiation in an ICOS-independent manner^121^, consistent with an earlier study showing that the constitutively active form of PI3K subunit p110α was also sufficient to increase Tfh cell responses^122^. Interestingly, it seems that PI3K signaling is also used by PD-1, via interaction with bystander B cells expressing PD-L1, to regulate Tfh cell position in GCs^123^. Shi *et al*. showed that *in vitro*, PD-1 suppressed PI3K activation triggered by CXCR5, and *in vivo*, PD-1 antagonized ICOS and suppressed the follicle recruitment of Tfh cells^123^. Meanwhile, the PD-1 engagement promoted Tfh cell concentration in the GC by limiting CXCR3-mediated distraction^123^.

Following the idea that a contact-based mechanism may be critical for Tfh cell trafficking and retention in GCs, Lu *et al*. screened the erythropoietin-producing hepatocellular receptor (EPH) and EPH-interacting protein (ephrin, EFN) family^124^. They found that the deficiency of EFNB1, a class B ephrin highly expressed in GC B cells, and the knockdown of EPHB6, a class B EPH receptor induced in Tfh cells and bound by EFNB1, both resulted in exaggerated GC Tfh retention^124^. Although EFNB1 did not seem to affect the overall GC B cell responses, its deficiency impacted the plasma cell formation, particularly the bone marrow plasma cell compartment^124^. They also found that EFNB1 and EPHB4 were necessary for IL-21 production by Tfh cells^124^.

Another interesting pair of contact-dependent adhesive guidance receptors for Tfh cells in GC responses are Plexin B2 (PlxnB2) and Sema4C^125^. Similar to EFNB1 and EPHBs, PlxnB2 was uniquely expressed on GC B cells whereas Sema4C was specifically expressed on Tfh cells^125^. Yan *et al*. showed that in PlxnB2-deficient GCs, the Tfh cells were concentrated along the edge of the GC^125^. The disruption of PlxnB2-Sema4C interactions interfered with the directional guidance of Tfh cells deep into the GC and reduced antibody affinity maturation and generation of plasma cells^125^.

A separate study showed that α_V_ integrin was also essential for the accumulation of Tfh cells in the GC^126^. The study found that α_V_ integrin was not required for Tfh cell differentiation or provision of help to B cells; rather, α_V_ integrin was important for Tfh accumulation in GCs^126^. Although early antibody responses were normal, the deletion of α_V_ integrin resulted in reduced number and size of GCs at later time points of the response to protein immunization, and the generation of long-lived antibody-producing cells in influenza virus infection model was compromised^126^. Interestingly, in the absence of α_V_ integrin, the generation of memory B cells seemed to be intact^126^, consistent with a study showing that memory B cells are generated in earlier waves than plasma cells during the GC responses^127^.

## Tfh cell metabolism

T cell activation and differentiation are accompanied by dynamic metabolic re-programming^128,129^. The mechanistic target of rapamycin (mTOR) is a serine/threonine kinase that senses and integrates multiple signals to regulate cell growth, proliferation, differentiation, metabolism, and survival^130,131^. Interacting with scaffold proteins Raptor and Rictor, mTOR forms two distinct complexes—mTORC1 and mTORC2, respectively—that have different sensitivities to rapamycin as well as upstream signals and downstream functions^130,131^.

An early study by Ray *et al*. on mTOR signaling in Tfh cells showed that in the LCMV model, compared with Th1 cells, Tfh cells were less proliferative and had less metabolic function, including less glycolysis and mitochondrial metabolism^132^. Using a short hairpin RNA (shRNA) knockdown approach, the authors showed that mTOR silencing in activated SMARTA T cells led to reduced Th1 cell differentiation but did not affect Bcl6 expression or Tfh cell differentiation^132^. In the LCMV model, previous studies have shown that activated Blimp1^+^CD4^+^ T cells expressed high levels of CD25^63^. Combined with the results that Tfh cells had reduced Akt and mTOR signaling compared with Th1 cells *in vivo* and that overexpression of constitutively active Akt promoted Th1 cell differentiation, that study suggests that in addition to the IL-2/STAT5/Blimp1 axis, the IL-2/Akt/mTOR axis also favors Th1 cell development at the expense of Tfh cells^132^.

However, three independent studies using Raptor- and Rictor-conditional KO mouse lines reached a different conclusion. (1) Zeng *et al*. showed that ICOS stimulation activated mTORC1 and mTORC2 and promoted glucose uptake, glycolysis, and lipogenesis for Tfh cell differentiation^122^. Interestingly, mTORC1 and mTORC2 induced discrete programs, in which mTORC2 regulated the activation and translocation of Foxo1^122^, an important transcription factor that has been shown to be important for Tfh cell development^80^. (2) Yang *et al*. showed that whereas mTORC1-deficient CD4^+^ T cells were severely defective in cell proliferation and Tfh cell differentiation^67^, mTORC2-mediated control of Tfh cell differentiation was partially attributed to its regulation of Akt activation and TCF1/Bcl6 axis^67^. (3) Hao *et al*. confirmed that TCR and ICOS stimulation activated mTORC2 signaling and that mTORC2 was important for Tfh cell differentiation after the early stage of the CD4^+^ T cell response^133^. Additionally, the study found that mTORC2 deficiency led to impaired cell migration and B cell help function^133^. Although there were a few discrepancies among these three studies—regarding cell proliferation/survival, Bcl6 and TCF1 expression levels, and so on^67,122,133^—the collective results were in agreement that both mTORC1 and mTORC2 intrinsically promoted Tfh cell development.

The opposite conclusions on the role of mTOR signaling in Tfh cell differentiation may stem from the experimental approaches used: Ray *et al*. took the shRNA knockdown approach in activated CD4^+^ T cells^132^, whereas the other three groups studied genetic deletion in naïve CD4^+^ T cells^67,122,133^. Interestingly, the published data regarding Tfh cell proliferation have also drawn opposite conclusions: some studies found that Tfh cells were less proliferative than Th1 cells^132^, whereas other studies reported that Bcl6^hi^ cells had higher proliferation and higher levels of cell cycle-related gene expression^58,134,135^. Upon further analysis, however, all these discrepancies are pointing to a more fundamental issue that needs to be addressed in the Tfh field: *in combination with timeline, can we identify more cell surface markers or new means to distinguish the various stages of Tfh cell differentiation and the subsets of Tfh cells?*

In most of the literature, CXCR5 expression has become an easy surrogate of Tfh cell differentiation. As mentioned above, studies have shown that CXCR5 expression was induced as early as the second or third cell division after infection^7,63,64^ or in activated CD4^+^ T cells that had proliferated most^66,67^. Meanwhile, a single-cell RNA sequencing analysis also suggests that the Th1 versus Tfh divergence occurs at a time point after the initial burst of cell proliferation^65^. Such early activated CXCR5^+^ “pre-Tfh” cells would appear to be highly proliferative. CXCR5^+^ pre-Tfh cells need to enter B cell follicles and become CXCR5^hi^ GC-Tfh cells to form GC responses^7,17,136^. There is evidence that after the early activation and expansion stage but before the full formation of the GC, CXCR5^+^ pre-Tfh cells have already reduced cell proliferation; that is, they are less proliferative^134^ (F.M. Zhu and H. Hu, unpublished data). Although many studies do not separate CXCR5^+^ pre-Tfh cells from CXCR5^hi^ GC-Tfh cells in their analyses, most CXCR5^hi^ GC-Tfh cells have been shown to be in a non-proliferative state^136^ (F.M. Zhu and H. Hu, unpublished data). The overexpression of Bcl6 resulted in the reduction of glucose metabolism of activated CD4^+^ T cells^137^. A separate study suggests that Bcl6 protein synthesis is also controlled by mTOR signaling^138^. Thus, it is conceivable that whereas PI3K-mTOR signaling supports the activation of CD4^+^ T cells and the differentiation of early-stage pre-Tfh cells (the highly proliferative ones) via TCF1- and Foxo1-mediated regulation, the progressively increased Bcl6 expression may reprogram the Tfh cells into a different metabolic state afterwards. Taken together, these studies indicate that mTOR signaling and metabolic requirements likely differ between the stages of Tfh cell differentiation. Thus, a more accurate understanding of the stepwise Tfh cell differentiation and the identification of stage-specific markers remain important pursuits in the Tfh field.

It has been reported that the microenvironment in GCs is hypoxic, and hypoxia-inducible factors (HIFs) have been shown to be critical for GC B cell responses^139^. In CD4^+^ T cells upon activation, Cho *et al*. showed that both HIF-1α and HIF-2α expression levels were regulated by mTORC1 and mTORC2 signaling and that the deletion of HIF-1α alone or both HIF-1α and HIF-2α led to the reduced generation of Tfh cells and an increased ratio of T follicular regulatory (Tfr) cells to Tfh cells^140^. Furthermore, HIF-regulated cytokines and cytokine-mediated metabolism influenced the function of Tfh cells providing help to B cells^140^.

Hypoxia-adenosinergic immunosuppression refers to the suppression of activated immune cells by hypoxia-driven accumulation of extracellular adenosine via cell surface A2 adenosine receptors^141^. The potential hypoxic environment in the GC led to the examination of the A2a adenosine receptor (A2aR) in Tfh cells, and indeed the deletion of A2aR in CD4^+^ T cells resulted in increased Tfh cell differentiation^142^. Similarly, as dephosphorylation of extracellular ATP generates extracellular adenosine^143^, ATP-gated ionotropic P2X7 receptor (P2RX7) has also been studied in Tfh responses^144^. Expressed at high levels in Tfh cells, P2RX7 has been found to mediate Tfh cell death and limit GC responses in Peyer’s patches^144^.

## Tfh cell number

A key feature of the GC B cell response is affinity maturation^145^. Using an innovative αDEC model system in which OVA antigen was delivered directly to GC B cells, Victora *et al*. showed that with increased opportunity to present antigens to Tfh cells and receive Tfh cell help, GC B cells had less competition with each other, leading to a decrease in the number of high-affinity B cells^146^. The study suggested that T cell help is a limiting factor for affinity maturation^146^. These observations have raised intriguing questions of whether Tfh cell number is a limiting factor and how the manipulation of the number of Tfh cells—and thereby the help provided by these cells—may shape the GC responses and affinity selection.

Previous studies have shown that deletion of transcription factor Foxp1 leads to dramatically enhanced Tfh cell responses, GC B cell responses, and the production of antibodies, including high-affinity antibodies^79^. A recent mechanistic study revealed that part of the Foxp1-mediated regulation of Tfh cell differentiation is through CTLA-4: CTLA-4 is a direct target of Foxp1, CTLA-4 expression levels were decreased in the absence of Foxp1, and overexpression of CTLA-4 suppressed Tfh cell differentiation^68^. Deleting Foxp1 in CD4^+^ T cells or blocking CTLA-4 led to increased numbers of Tfh cells, and both situations resulted in increased GC B cell numbers and selection of high-affinity B cells as well as abolished intra-clonal B cell competition^68^, as high numbers of single-clone B cells have been shown to result in intra-clonal B cell competition and reduced affinity maturation^147^. Other studies have also shown that increasing or decreasing the number of Tfh cells—through genetic manipulation or chemical intervention—increases or decreases the GC B cell responses accordingly^92,148–150^.

To summarize, the help provided by Tfh cells during Tfh-GC B interaction is a limiting resource during the selection process in the GC; meanwhile, the collective results support the notion that increased Tfh numbers lead to stronger GC responses, resulting in more GC B cells, increased BCR affinity, and higher antibody titer. So far none of the existing model systems have separated a change in Tfh cell number from Tfh cell function. Manipulating Tfh cell number and function will continue to be important topics for studies of the GC response and vaccine development/vaccination strategies.

## Tfh subsets

Studies have shown that during GC responses, activated GC B cells have multiple fates^145^. This observation naturally leads to an intriguing question of whether there are different Tfh subsets with varied functions to help B cells. IL-21 and IL-4 are two well-recognized cytokines produced by Tfh cells providing help to B cells^10,11,151–155^. Using IL-21 and IL-4 dual-reporter mice in a subcutaneous helminth infection model, Weinstein *et al*. showed that these two important cytokines were produced in a progressive manner by Tfh cells^156^. IL-21 was generated first in the early time points of the response, followed by IL-4 production and a substantial number of double-positive cells^156^. At the later stage, IL-21 production was gradually lost but IL-4 expression was sustained^156^. The cytokine-based Tfh subsets—termed Tfh21, Tfh21+4, and Tfh4 cells—were found to have different transcriptome profiling, and Tfh21 and Tfh4 localized differently in the GC^156^. The three groups of Tfh cells also displayed distinct functions in helping B cells: Tfh21 cells seemed to assist more in promoting high-affinity selection whereas Tfh21+4 and Tfh4 cells facilitated the generation of plasma cells^156^.

Besides Tfh21, Tfh21+4, and Tfh4 cells, Gowthaman *et al*. recently identified an IL-13-producing Tfh subset (Tfh13) that drives the production of high-affinity IgE antibodies^157^. Previously, it was shown that IL-4 production in Tfh cells is independent of Gata3^158^—an essential transcription factor for IL-4 production in Th2 cells^159^. While studying a hyper-IgE phenotype associated with Dock8 deficiency, Gowthaman *et al*. found that a subset of Dock8-deficient Tfh cells expressed high levels of IL-4, IL-5, and IL-13 but low levels of IL-21^157^. This unique Tfh13 subset was also induced in wild-type mice during allergic sensitization to multiple allergens but not in a murine helminth infection model of Th2 responses^157^. More importantly, Tfh13 cells were also detected in the peripheral blood of patients with a peanut allergy^157^. In an independent study of a house dust mite sensitization model, IL-13-producing Tfh cells were also observed^160^.

In the CD4^+^ T cell responses to Zika virus (ZIKV), a preferential Th1-like Tfh cell response has been observed^161^. The robust interferon gamma (IFNγ)-producing Tfh cells were induced by replicable ZIKV infection, and the Th1-like Tfh cell differentiation was T-bet-dependent^161^. IFNγ-producing Tfh cells were also found in responses to LCMV infection^162–164^. Interestingly, depending on the environment setting, T-bet actually plays diversified roles in Tfh cell differentiation and function. In the CD4^+^ T cell responses to influenza infection, Sheikh *et al*. showed that the loss of T-bet promoted Tfh cell differentiation at the expense of Th1 cells^163^. Supporting this, in a *Salmonella* infection model, Elsner *et al*. showed that the *Salmonella* infection induced IL-12 production, which in turn induced high levels of T-bet that favored Th1 cell differentiation and suppressed Tfh cell differentiation^165^. In LCMV infection, however, under T cell competition conditions, the loss of T-bet resulted in a deficiency of both Th1 and Tfh cell responses^162,163^. For Th1-like Tfh cells, using a T-bet fate-mapping mouse strain, Fang *et al*. reported that the IFNγ-producing GC-Tfh subset had a history of T-bet expression^166^. Within the GC-Tfh cell population, all the cells capable of IFNγ production were from this Th1-like Tfh subset; the *Ifng* locus was found to be partially accessible only in such cells, and the early stage of T-bet expression was essential^166^.

Regarding IL-2 production by Tfh cells, Papillion *et al*. recently showed that GC-Tfh cells secreted large amounts of IL-2^167^. The study went further to link the compromised late Tfh cell responses in the absence of IL-6 to IL-2 responsiveness of GC-Tfh cells, such that IL-6 signaling restrained the suppressive function of IL-2 on GC-Tfh cells by reducing CD122 expression^167^.

It is very interesting that the cytokine profiles of Tfh cells seem to be in accord with the infection to which they are responding, and these subsets may localize differently within the GC microanatomy and help B cells differently. Further understanding of the underlying mechanism will generate new opportunities for the fine tuning of the GC response in the treatment of diseases or in prophylactic vaccines.

## Tfh memory

The recall responses of CXCR5^+^CD4^+^ memory T cells in various model systems appear to be quite different. Studies in protein vaccination and LCMV infection models have shown that CXCR5^+^CD4^+^ Tfh memory cells form after the primary CD4^+^ T cell responses, and they preferentially give rise to Tfh recall responses^168,169^. Recently, it was reported that both local and circulating Tfh memory cells function in helping the secondary humoral responses in mice^170^. On the other hand, in a *Listeria monocytogenes* infection model, Pepper *et al*. showed that the CXCR5^+^CD4^+^ memory T cells adopted a central memory T cell phenotype as they expressed CCR7 and were located mainly in T cell areas after transfer^18^. Upon re-challenge, CXCR5^+^CD4^+^ memory T cells were less potent in IFNγ production compared with Th1 effector memory T cells, but they rapidly produced IL-2 and generated a diverse secondary response, including robust non-Tfh responses^18^. In this regard, the mixed/diverse recall responses of CXCR5^+^CD4^+^ memory T cells suggest multi-potency or plasticity.

Recently, in addition to having an important role in Tfh cell differentiation^112^, the transcription factor Thpok has been found to be critical for central memory CD4^+^ T cells^171^. Using single-cell RNA sequencing, Ciucci *et al*. identified a unique gene expression signature that excludes typical Tfh and Th1 effector genes but contains genes linked to memory potential^171^. These CD4^+^ T cells expressed CXCR5 and CCR7 and were designated T central memory precursors^171^. Thpok-deficient CD4^+^ T cells in the memory stage exhibited lower levels of *Cxcr5*, *Ccr7*, *Tcf7*, *Bcl2*, and *Il7R*; mechanistically, Thpok was found to be essential for the generation and functional fitness of memory precursors by directly repressing *Prdm1* and *Runx3* expression^171^.

After naïve CD4^+^ T cells are activated, presumably not all the newly generated CXCR5^+^CD4^+^ T cells at the T-B border will enter B cell follicles and become GC-Tfh cells. The studies mentioned above raise the question of which CXCR5^+^CD4^+^ T cells would differentiate into PD-1^hi^CXCR5^hi^ GC-Tfh cells versus CXCR5^+^CD4^+^ “central” memory T cells.

CXCR5^+^CD4^+^ memory T cells have been shown to be multi-potent^18^. By blocking cell death induced by NAD-mediated ribosylation of P2RX7 during isolation from tissues, in the LCMV infection model, Kunzli *et al*. showed that long-lived Tfh cells retained plasticity^172^. In their study, compared with day 15 GC-Tfh cells, the long-lived (>400 days) Tfh cells expressed PD-1 and CXCR5 at levels resembling CXCR5^+^CD4^+^ memory T cells^172^. It has been suggested that when GC response was fading, PD-1^hi^CXCR5^hi ^GC-Tfh cells downregulated Bcl6 and CXCR5 expression levels^134,136^. Thus, whereas the CXCR5^+^CD4^+^ memory T cell population as a whole can give rise to both non-Tfh and Tfh responses^18^, it is unclear whether this is due to the plasticity of these cells or because CXCR5^+^CD4^+^ memory cells represent a heterogeneous population of *bona fide* GC-Tfh memory cells mixed with less differentiated “central plastic” memory cells. The differences and relationship between CXCR5^+^CD4^+^CCR7^–^ pre-Tfh cells, CXCR5^+^CD4^+^CCR7^+^ “central” memory precursors, and CXCR5^hi^ GC-Tfh cells are still under-studied and remain important issues that need to be addressed.

## Concluding remarks

Tfh cells are essential for GC B cell responses and antibody affinity maturation. As most of the successful vaccines function by inducing protective antibody responses to T-dependent antigens, the differentiation and function of Tfh cells will continue to be crucial topics in vaccine design. Recently, a study in non-human primates demonstrated that low-dose immunizations over prolonged intervals led to enhanced antibody responses to HIV antigens, and part of the mechanism was an increase in Tfh cells targeting a broader set of epitopes^173^. That study provides one example of continued efforts to generate novel vaccines and the crucial role played by Tfh cells in the process. Additionally, Tfh cells have been shown to be associated with a broad range of autoimmune diseases^5,38^, suggesting that discoveries in this field will have wide-reaching implications.

There are many other new and exciting findings regarding Tfh and GC responses—including Tfr cells, follicular cytotoxic T (Tfc) cells, miRNA regulation of Tfh cells, the human Tfh studies, and Tfh cells in diseases—that due to space constraints could not be included in this limited review. Questions remain regarding our understanding of the mechanism of Tfh cell differentiation, Tfh subsets, memory, and regulation of Tfh function. Future studies will help us take advantage of this unique population of helper CD4^+^ cells for vaccine development and the treatment of diseases.

## Abbreviations

A2aR, A2a adenosine receptor; APC, antigen-presenting cell; cDC, conventional dendritic cell; cDC1, type 1 conventional dendritic cell; cDC2, type 2 conventional dendritic cell; DC, dendritic cell; DT, diphtheria toxin; EFN, EPH-interacting protein; EPH, erythropoietin-producing hepatocellular receptor; GC, germinal center; GC-Tfh, germinal center Tfh; HLH, helix-loop-helix; HMT, histone methyltransferase; KO, knockout; mTOR, mechanistic target of rapamycin; P2RX7, ATP-gated ionotropic P2X7 receptor; pDC, plasmacytoid dendritic cell; PD-L1, PD-1 ligand; PI3Kδ, PI3K subunit p110δ; pre-Tfh, precursor Tfh; TCR, T-cell receptor; Tfc, T follicular cytotoxic; Tfh, T follicular helper; Tfh13, IL-13-producing Tfh; Tfr, T follicular regulatory; zDC, transcription factor Zbtb46

## References

[1] Zhu J Yamane H Paul WE: Differentiation of effector CD4 T cell populations (*). *Annu Rev Immunol.* 2010; 28: 445–89. 10.1146/annurev-immunol-030409-101212 20192806 PMC3502616

[2] Breitfeld D Ohl L Kremmer E: Follicular B helper T cells express CXC chemokine receptor 5, localize to B cell follicles, and support immunoglobulin production. *J Exp Med.* 2000; 192(11): 1545–52. 10.1084/jem.192.11.1545 11104797 PMC2193094

[3] Schaerli P Willimann K Lang AB: CXC chemokine receptor 5 expression defines follicular homing T cells with B cell helper function. *J Exp Med.* 2000; 192(11): 1553–62. 10.1084/jem.192.11.1553 11104798 PMC2193097

[4] Crotty S: Follicular helper CD4 T cells (TFH). *Annu Rev Immunol.* 2011; 29: 621–63. 10.1146/annurev-immunol-031210-101400 21314428

[5] Vinuesa CG Linterman MA Di Yu: Follicular Helper T Cells. *Annu Rev Immunol.* 2016; 34: 335–68. 10.1146/annurev-immunol-041015-055605 26907215

[6] Cannons JL Qi H Lu KT: Optimal germinal center responses require a multistage T cell:B cell adhesion process involving integrins, SLAM-associated protein, and CD84. *Immunity.* 2010; 32(2): 253–65. 10.1016/j.immuni.2010.01.010 20153220 PMC2830297

[7] Choi YS Kageyama R Eto D: ICOS receptor instructs T follicular helper cell versus effector cell differentiation via induction of the transcriptional repressor Bcl6. *Immunity.* 2011; 34(6): 932–46. 10.1016/j.immuni.2011.03.023 21636296 PMC3124577

[8] Kerfoot SM Yaari G Patel JR: Germinal center B cell and T follicular helper cell development initiates in the interfollicular zone. *Immunity.* 2011; 34(6): 947–60. 10.1016/j.immuni.2011.03.024 21636295 PMC3280079

[9] Goenka R Barnett LG Silver JS: Cutting edge: Dendritic cell-restricted antigen presentation initiates the follicular helper T cell program but cannot complete ultimate effector differentiation. *J Immunol.* 2011; 187(3): 1091–5. 10.4049/jimmunol.1100853 21715693 PMC3171798

[10] Nurieva RI Chung Y Hwang D: Generation of T follicular helper cells is mediated by interleukin-21 but independent of T helper 1, 2, or 17 cell lineages. *Immunity.* 2008; 29(1): 138–49. 10.1016/j.immuni.2008.05.009 18599325 PMC2556461

[11] Vogelzang A McGuire HM Di Yu: A fundamental role for interleukin-21 in the generation of T follicular helper cells. *Immunity.* 2008; 29(1): 127–37. 10.1016/j.immuni.2008.06.001 18602282

[12] Ma CS Suryani S Avery DT: Early commitment of naïve human CD4(+) T cells to the T follicular helper (T(FH)) cell lineage is induced by IL-12. *Immunol Cell Biol.* 2009; 87(8): 590–600. 10.1038/icb.2009.64 19721453

[13] Schmitt N Morita R Bourdery L: Human dendritic cells induce the differentiation of interleukin-21-producing T follicular helper-like cells through interleukin-12. *Immunity.* 2009; 31(1): 158–69. 10.1016/j.immuni.2009.04.016 19592276 PMC2731623

[14] Ballesteros-Tato A León B Graf BA: Interleukin-2 inhibits germinal center formation by limiting T follicular helper cell differentiation. *Immunity.* 2012; 36(5): 847–56. 10.1016/j.immuni.2012.02.012 22464171 PMC3361521

[15] Choi YS Eto D Yang JA: Cutting edge: STAT1 is required for IL-6-mediated Bcl6 induction for early follicular helper cell differentiation. *J Immunol.* 2013; 190(7): 3049–53. 10.4049/jimmunol.1203032 23447690 PMC3626564

[16] Johnston RJ Choi YS Diamond JA: STAT5 is a potent negative regulator of TFH cell differentiation. *J Exp Med.* 2012; 209(2): 243–50. 10.1084/jem.20111174 22271576 PMC3281266

[17] Xu H Li X Liu D: Follicular T-helper cell recruitment governed by bystander B cells and ICOS-driven motility. *Nature.* 2013; 496(7446): 523–7. 10.1038/nature12058 23619696

[18] Pepper M Pagán AJ Igyártó BZ: Opposing signals from the Bcl6 transcription factor and the interleukin-2 receptor generate T helper 1 central and effector memory cells. *Immunity.* 2011; 35(4): 583–95. 10.1016/j.immuni.2011.09.009 22018468 PMC3208313

[19] Banchereau J Briere F Caux C: Immunobiology of dendritic cells. *Annu Rev Immunol.* 2000; 18: 767–811. 10.1146/annurev.immunol.18.1.767 10837075

[20] Eisenbarth SC: Dendritic cell subsets in T cell programming: Location dictates function. *Nat Rev Immunol.* 2019; 19(2): 89–103. 10.1038/s41577-018-0088-1 30464294 PMC7755085

[21] Krishnaswamy JK Alsén S Yrlid U: Determination of T Follicular Helper Cell Fate by Dendritic Cells. *Front Immunol.* 2018; 9: 2169. 10.3389/fimmu.2018.02169 30319629 PMC6170619

[22] Schlitzer A Zhang W Song M: Recent advances in understanding dendritic cell development, classification, and phenotype [version 1; peer review: 2 approved]. *F1000Res.* 2018; 7: F1000 Faculty Rev-1558. 10.12688/f1000research.14793.1 30345015 PMC6173131

[23] Meredith MM Liu K Darrasse-Jeze G: Expression of the zinc finger transcription factor zDC (Zbtb46, Btbd4) defines the classical dendritic cell lineage. *J Exp Med.* 2012; 209(6): 1153–65. 10.1084/jem.20112675 22615130 PMC3371731

[24] Satpathy AT KC W Albring JC: Zbtb46 expression distinguishes classical dendritic cells and their committed progenitors from other immune lineages. *J Exp Med.* 2012; 209(6): 1135–52. 10.1084/jem.20120030 22615127 PMC3371733

[25] Woodruff MC Heesters BA Herndon CN: Trans-nodal migration of resident dendritic cells into medullary interfollicular regions initiates immunity to influenza vaccine. *J Exp Med.* 2014; 211(8): 1611–21. 10.1084/jem.20132327 25049334 PMC4113935

[26] Gerner MY Torabi-Parizi P Germain RN: Strategically localized dendritic cells promote rapid T cell responses to lymph-borne particulate antigens. *Immunity.* 2015; 42(1): 172–85. 10.1016/j.immuni.2014.12.024 25607462

[27] Krishnaswamy JK Gowthaman U Zhang B: Migratory CD11b+ conventional dendritic cells induce T follicular helper cell-dependent antibody responses. *Sci Immunol.* 2017; 2(18): eaam9169. 10.1126/sciimmunol.aam9169 29196450 PMC7847246

[28] Li J Lu E Yi T: EBI2 augments Tfh cell fate by promoting interaction with IL-2-quenching dendritic cells. *Nature.* 2016; 533(7601): 110–4. 10.1038/nature17947 27147029 PMC4883664

[29] Pereira JP Kelly LM Xu Y Cyster JG: EBI2 mediates B cell segregation between the outer and centre follicle. *Nature.* 2009; 460(7259): 1122–6. 10.1038/nature08226 19597478 PMC2809436

[30] Gatto D Paus D Basten A: Guidance of B cells by the orphan G protein-coupled receptor EBI2 shapes humoral immune responses. *Immunity.* 2009; 31(2): 259–69. 10.1016/j.immuni.2009.06.016 19615922

[31] Arroyo EN Pepper M: B cells are sufficient to prime the dominant CD4+ Tfh response to Plasmodium infection. *J Exp Med.* 2020; 217(2): e20190849. 10.1084/jem.20190849 31748243 PMC7041722

[32] Dahlgren MW Gustafsson-Hedberg T Livingston M: T follicular helper, but not Th1, cell differentiation in the absence of conventional dendritic cells. *J Immunol.* 2015; 194(11): 5187–99. 10.4049/jimmunol.1401938 25917099

[33] Benson RA MacLeod MKL Hale BG: Antigen presentation kinetics control T cell/dendritic cell interactions and follicular helper T cell generation in vivo. *eLife.* 2015; 4: e06994. 10.7554/eLife.06994 26258879 PMC4558563

[34] Kumamoto Y Hirai T Wong PW: CD301b+ dendritic cells suppress T follicular helper cells and antibody responses to protein antigens. *Elife.* 2016; 5: e17979. 10.7554/eLife.17979 27657168 PMC5033605

[35] Hardtke S Ohl L Förster R: Balanced expression of CXCR5 and CCR7 on follicular T helper cells determines their transient positioning to lymph node follicles and is essential for efficient B-cell help. *Blood.* 2005; 106(6): 1924–31. 10.1182/blood-2004-11-4494 15899919

[36] Haynes NM Allen CDC Lesley R: Role of CXCR5 and CCR7 in follicular Th cell positioning and appearance of a programmed cell death gene-1high germinal center-associated subpopulation. *J Immunol.* 2007; 179(8): 5099–108. 10.4049/jimmunol.179.8.5099 17911595

[37] Martinez GJ Hu JK Pereira RM: Cutting Edge: NFAT Transcription Factors Promote the Generation of Follicular Helper T Cells in Response to Acute Viral Infection. *J Immunol.* 2016; 196(5): 2015–9. 10.4049/jimmunol.1501841 26851216 PMC4761453

[38] Crotty S: T follicular helper cell differentiation, function, and roles in disease. *Immunity.* 2014; 41(4): 529–42. 10.1016/j.immuni.2014.10.004 25367570 PMC4223692

[39] Liu X Chen X Zhong B: Transcription factor achaete-scute homologue 2 initiates follicular T-helper-cell development. *Nature.* 2014; 507(7493): 513–8. 10.1038/nature12910 24463518 PMC4012617

[40] Shaw LA Bélanger S Omilusik KD: Id2 reinforces TH1 differentiation and inhibits E2A to repress TFH differentiation. *Nat Immunol.* 2016; 17(7): 834–43. 10.1038/ni.3461 27213691 PMC4915968

[41] Muto A Tashiro S Nakajima O: The transcriptional programme of antibody class switching involves the repressor Bach2. *Nature.* 2004; 429(6991): 566–71. 10.1038/nature02596 15152264

[42] Roychoudhuri R Hirahara K Mousavi K: BACH2 represses effector programs to stabilize T_reg_-mediated immune homeostasis. *Nature.* 2013; 498(7455): 506–10. 10.1038/nature12199 23728300 PMC3710737

[43] Kim EH Gasper DJ Lee SH: Bach2 regulates homeostasis of Foxp3^+^ regulatory T cells and protects against fatal lung disease in mice. *J Immunol.* 2014; 192(3): 985–95. 10.4049/jimmunol.1302378 24367030 PMC3946995

[44] Tsukumo S-i Unno M Muto A: Bach2 maintains T cells in a naive state by suppressing effector memory-related genes. *Proc Natl Acad Sci U S A.* 2013; 110(26): 10735–40. 10.1073/pnas.1306691110 23754397 PMC3696756

[45] Roychoudhuri R Clever D Li P: BACH2 regulates CD8(+) T cell differentiation by controlling access of AP-1 factors to enhancers. *Nat Immunol.* 2016; 17(7): 851–60. 10.1038/ni.3441 27158840 PMC4918801

[46] Kuwahara M Ise W Ochi M: Bach2-Batf interactions control Th2-type immune response by regulating the IL-4 amplification loop. *Nat Commun.* 2016; 7: 12596. 10.1038/ncomms12596 27581382 PMC5025763

[47] Ochiai K Katoh Y Ikura T: Plasmacytic transcription factor Blimp-1 is repressed by Bach2 in B cells. *J Biol Chem.* 2006; 281(50): 38226–34. 10.1074/jbc.M607592200 17046816

[48] Ochiai K Muto A Tanaka H: Regulation of the plasma cell transcription factor Blimp-1 gene by Bach2 and Bcl6. *Int Immunol.* 2008; 20(3): 453–60. 10.1093/intimm/dxn005 18256039

[49] Lahmann A Kuhrau J Fuhrmann F: Bach2 Controls T Follicular Helper Cells by Direct Repression of Bcl-6. *J Immunol.* 2019; 202(8): 2229–39. 10.4049/jimmunol.1801400 30833348

[50] Geng J Wei H Shi B: Bach2 Negatively Regulates T Follicular Helper Cell Differentiation and Is Critical for CD4^+^ T Cell Memory. *J Immunol.* 2019; 202(10): 2991–8. 10.4049/jimmunol.1801626 30971440 PMC6504585

[51] Zhang H Hu Q Zhang M: Bach2 Deficiency Leads to Spontaneous Expansion of IL-4-Producing T Follicular Helper Cells and Autoimmunity. *Front Immunol.* 2019; 10: 2050. 10.3389/fimmu.2019.02050 31552021 PMC6737000

[52] Leong YA Chen Y Ong HS: CXCR5(+) follicular cytotoxic T cells control viral infection in B cell follicles. *Nat Immunol.* 2016; 17(10): 1187–96. 10.1038/ni.3543 27487330

[53] Arnold CN Campbell DJ Lipp M: The germinal center response is impaired in the absence of T cell-expressed CXCR5. *Eur J Immunol.* 2007; 37(1): 100–9. 10.1002/eji.200636486 17171760

[54] Vanderleyden I Fra-Bido SC Innocentin S: Follicular Regulatory T Cells Can Access the Germinal Center Independently of CXCR5. *Cell Rep.* 2020; 30(3): 611–619.e4. 10.1016/j.celrep.2019.12.076 31968240 PMC6988108

[55] Fazilleau N McHeyzer-Williams LJ Rosen H: The function of follicular helper T cells is regulated by the strength of T cell antigen receptor binding. *Nat Immunol.* 2009; 10(4): 375–84. 10.1038/ni.1704 19252493 PMC2712297

[56] Tubo NJ Pagán AJ Taylor JJ: Single naive CD4^+^ T cells from a diverse repertoire produce different effector cell types during infection. *Cell.* 2013; 153(4): 785–96. 10.1016/j.cell.2013.04.007 23663778 PMC3766899

[57] Baumjohann D Preite S Reboldi A: Persistent antigen and germinal center B cells sustain T follicular helper cell responses and phenotype. *Immunity.* 2013; 38(3): 596–605. 10.1016/j.immuni.2012.11.020 23499493

[58] DiToro D Winstead CJ Pham D: Differential IL-2 expression defines developmental fates of follicular versus nonfollicular helper T cells. *Science.* 2018; 361(6407): eaao2933. 10.1126/science.aao2933 30213884 PMC6501592

[59] Keck S Schmaler M Ganter S: Antigen affinity and antigen dose exert distinct influences on CD4 T-cell differentiation. *Proc Natl Acad Sci U S A.* 2014; 111(41): 14852–7. 10.1073/pnas.1403271111 25267612 PMC4205596

[60] Kotov DI Mitchell JS Pengo T: TCR Affinity Biases Th Cell Differentiation by Regulating CD25, Eef1e1, and Gbp2. *J Immunol.* 2019; 202(9): 2535–45. 10.4049/jimmunol.1801609 30858199 PMC6478541

[61] Matsuyama T Grossman A Mittrücker HW: Molecular cloning of *LSIRF*, a lymphoid-specific member of the interferon regulatory factor family that binds the interferon-stimulated response element (ISRE). *Nucleic Acids Res.* 1995; 23(12): 2127–36. 10.1093/nar/23.12.2127 7541907 PMC306999

[62] Krishnamoorthy V Kannanganat S Maienschein-Cline M: The IRF4 Gene Regulatory Module Functions as a Read-Write Integrator to Dynamically Coordinate T Helper Cell Fate. *Immunity.* 2017; 47(3): 481–497.e7. 10.1016/j.immuni.2017.09.001 28930660 PMC5661949

[63] Wu T Shin HM Moseman EA: TCF1 Is Required for the T Follicular Helper Cell Response to Viral Infection. *Cell Rep.* 2015; 12(12): 2099–110. 10.1016/j.celrep.2015.08.049 26365183 PMC4591235

[64] Chen X Cao G Wu J: The histone methyltransferase EZH2 primes the early differentiation of follicular helper T cells during acute viral infection. *Cell Mol Immunol.* 2020; 17(3): 247–60. 10.1038/s41423-019-0219-z 30842630 PMC7052164

[65] Lönnberg T Svensson V James KR: Single-cell RNA-seq and computational analysis using temporal mixture modelling resolves Th1/Tfh fate bifurcation in malaria. *Sci Immunol.* 2017; 2(9): eaal2192. 10.1126/sciimmunol.aal2192 28345074 PMC5365145

[66] Baumjohann D Okada T Ansel KM: Cutting Edge: Distinct waves of BCL6 expression during T follicular helper cell development. *J Immunol.* 2011; 187(5): 2089–92. 10.4049/jimmunol.1101393 21804014

[67] Yang J Lin X Pan Y: Critical roles of mTOR Complex 1 and 2 for T follicular helper cell differentiation and germinal center responses. *Elife.* 2016; 5: e17936. 10.7554/eLife.17936 27690224 PMC5063587

[68] Shi B Geng J Wang YH: Foxp1 Negatively Regulates T Follicular Helper Cell Differentiation and Germinal Center Responses by Controlling Cell Migration and CTLA-4. *J Immunol.* 2018; 200(2): 586–94. 10.4049/jimmunol.1701000 29212910 PMC5891213

[69] Johnston RJ Poholek AC DiToro D: Bcl6 and Blimp-1 are reciprocal and antagonistic regulators of T follicular helper cell differentiation. *Science.* 2009; 325(5943): 1006–10. 10.1126/science.1175870 19608860 PMC2766560

[70] Nurieva RI Chung Y Martinez GJ: Bcl6 mediates the development of T follicular helper cells. *Science.* 2009; 325(5943): 1001–5. 10.1126/science.1176676 19628815 PMC2857334

[71] Di Yu Rao S Tsai LM: The transcriptional repressor Bcl-6 directs T follicular helper cell lineage commitment. *Immunity.* 2009; 31(3): 457–68. 10.1016/j.immuni.2009.07.002 19631565

[72] Betz BC Jordan-Williams KL Wang C: Batf coordinates multiple aspects of B and T cell function required for normal antibody responses. *J Exp Med.* 2010; 207(5): 933–42. 10.1084/jem.20091548 20421391 PMC2867277

[73] Bauquet AT Jin H Paterson AM: The costimulatory molecule ICOS regulates the expression of c-Maf and IL-21 in the development of follicular T helper cells and TH-17 cells. *Nat Immunol.* 2009; 10(2): 167–75. 10.1038/ni.1690 19098919 PMC2742982

[74] Kwon H Thierry-Mieg D Thierry-Mieg J: Analysis of interleukin-21-induced Prdm1 gene regulation reveals functional cooperation of STAT3 and IRF4 transcription factors. *Immunity.* 2009; 31(6): 941–52. 10.1016/j.immuni.2009.10.008 20064451 PMC3272079

[75] Ise W Kohyama M Schraml BU: The transcription factor BATF controls the global regulators of class-switch recombination in both B cells and T cells. *Nat Immunol.* 2011; 12(6): 536–43. 10.1038/ni.2037 21572431 PMC3117275

[76] Bollig N Brüstle A Kellner K: Transcription factor IRF4 determines germinal center formation through follicular T-helper cell differentiation. *Proc Natl Acad Sci U S A.* 2012; 109(22): 8664–9. 10.1073/pnas.1205834109 22552227 PMC3365194

[77] Auderset F Schuster S Fasnacht N: Notch signaling regulates follicular helper T cell differentiation. *J Immunol.* 2013; 191(5): 2344–50. 10.4049/jimmunol.1300643 23918982

[78] Omodho B Miao T Symonds ALJ: Transcription factors early growth response gene (Egr) 2 and 3 control inflammatory responses of tolerant T cells. *Immun Inflamm Dis.* 2018; 6(2): 221–33. 10.1002/iid3.210 29314730 PMC5946152

[79] Wang H Geng J Wen X: The transcription factor Foxp1 is a critical negative regulator of the differentiation of follicular helper T cells. *Nat Immunol.* 2014; 15(7): 667–75. 10.1038/ni.2890 24859450 PMC4142638

[80] Stone EL Pepper M Katayama CD: ICOS coreceptor signaling inactivates the transcription factor FOXO1 to promote Tfh cell differentiation. *Immunity.* 2015; 42(2): 239–51. 10.1016/j.immuni.2015.01.017 25692700 PMC4334393

[81] Lee JY Skon CN Lee YJ: The transcription factor KLF2 restrains CD4^+^ T follicular helper cell differentiation. *Immunity.* 2015; 42(2): 252–64. 10.1016/j.immuni.2015.01.013 25692701 PMC4409658

[82] Weber JP Fuhrmann F Feist RK: ICOS maintains the T follicular helper cell phenotype by down-regulating Krüppel-like factor 2. *J Exp Med.* 2015; 212(2): 217–33. 10.1084/jem.20141432 25646266 PMC4322049

[83] Stauss D Brunner C Berberich-Siebelt F: The transcriptional coactivator Bob1 promotes the development of follicular T helper cells via Bcl6. *EMBO J.* 2016; 35(8): 881–98. 10.15252/embj.201591459 26957522 PMC4972135

[84] Serre K Mohr E Bénézech C: Selective effects of NF-κB1 deficiency in CD4^+^ T cells on Th2 and TFh induction by alum-precipitated protein vaccines. *Eur J Immunol.* 2011; 41(6): 1573–82. 10.1002/eji.201041126 21469117

[85] Weber BN Chi AWS Chavez A: A critical role for TCF-1 in T-lineage specification and differentiation. *Nature.* 2011; 476(7358): 63–8. 10.1038/nature10279 21814277 PMC3156435

[86] Yu S Zhou X Steinke FC: The TCF-1 and LEF-1 transcription factors have cooperative and opposing roles in T cell development and malignancy. *Immunity.* 2012; 37(5): 813–26. 10.1016/j.immuni.2012.08.009 23103132 PMC3501598

[87] Steinke FC Yu S Zhou X: TCF-1 and LEF-1 act upstream of Th-POK to promote the CD4(+) T cell fate and interact with Runx3 to silence *Cd4* in CD8(+) T cells. *Nat Immunol.* 2014; 15(7): 646–56. 10.1038/ni.2897 24836425 PMC4064003

[88] Jeannet G Boudousquié C Gardiol N: Essential role of the Wnt pathway effector Tcf-1 for the establishment of functional CD8 T cell memory. *Proc Natl Acad Sci U S A.* 2010; 107(21): 9777–82. 10.1073/pnas.0914127107 20457902 PMC2906901

[89] Zhou X Yu S Zhao DM: Differentiation and persistence of memory CD8(+) T cells depend on T cell factor 1. *Immunity.* 2010; 33(2): 229–40. 10.1016/j.immuni.2010.08.002 20727791 PMC2928475

[90] Yu Q Sharma A Oh SY: T cell factor 1 initiates the T helper type 2 fate by inducing the transcription factor GATA-3 and repressing interferon-gamma. *Nat Immunol.* 2009; 10(9): 992–9. 10.1038/ni.1762 19648923 PMC2824257

[91] Yu Q Sharma A Ghosh A: T cell factor-1 negatively regulates expression of IL-17 family of cytokines and protects mice from experimental autoimmune encephalomyelitis. *J Immunol.* 2011; 186(7): 3946–52. 10.4049/jimmunol.1003497 21339363 PMC3158594

[92] Choi YS Gullicksrud JA Xing S: LEF-1 and TCF-1 orchestrate T(FH) differentiation by regulating differentiation circuits upstream of the transcriptional repressor Bcl6. *Nat Immunol.* 2015; 16(9): 980–90. 10.1038/ni.3226 26214741 PMC4545301

[93] Xu L Cao Y Xie Z: The transcription factor TCF-1 initiates the differentiation of T(FH) cells during acute viral infection. *Nat Immunol.* 2015; 16(9): 991–9. 10.1038/ni.3229 26214740

[94] Shao P Li F Wang J: Cutting Edge: Tcf1 Instructs T Follicular Helper Cell Differentiation by Repressing Blimp1 in Response to Acute Viral Infection. *J Immunol.* 2019; 203(4): 801–6. 10.4049/jimmunol.1900581 31300510 PMC6684471

[95] Gullicksrud JA Li F Xing S: Differential Requirements for Tcf1 Long Isoforms in CD8^+^ and CD4^+^ T Cell Responses to Acute Viral Infection. *J Immunol.* 2017; 199(3): 911–9. 10.4049/jimmunol.1700595 28652395 PMC5531591

[96] Koyanagi M Baguet A Martens J: EZH2 and histone 3 trimethyl lysine 27 associated with *Il4* and *Il13* gene silencing in T_h_1 cells. *J Biol Chem.* 2005; 280(36): 31470–7. 10.1074/jbc.M504766200 16009709

[97] Tumes DJ Onodera A Suzuki A: The polycomb protein Ezh2 regulates differentiation and plasticity of CD4(+) T helper type 1 and type 2 cells. *Immunity.* 2013; 39(5): 819–32. 10.1016/j.immuni.2013.09.012 24238339

[98] Arvey A van der Veeken J Samstein RM: Inflammation-induced repression of chromatin bound by the transcription factor Foxp3 in regulatory T cells. *Nat Immunol.* 2014; 15(6): 580–7. 10.1038/ni.2868 24728351 PMC4112080

[99] DuPage M Chopra G Quiros J: The chromatin-modifying enzyme Ezh2 is critical for the maintenance of regulatory T cell identity after activation. *Immunity.* 2015; 42(2): 227–38. 10.1016/j.immuni.2015.01.007 25680271 PMC4347854

[100] Yang XP Jiang K Hirahara K: EZH2 is crucial for both differentiation of regulatory T cells and T effector cell expansion. *Sci Rep.* 2015; 5: 10643. 10.1038/srep10643 26090605 PMC4473539

[101] Li F Zeng Z Xing S: Ezh2 programs T_FH_ differentiation by integrating phosphorylation-dependent activation of Bcl6 and polycomb-dependent repression of p19Arf. *Nat Commun.* 2018; 9(1): 5452. 10.1038/s41467-018-07853-z 30575739 PMC6303346

[102] Long X Zhang L Zhang Y: Histone methyltransferase Nsd2 is required for follicular helper T cell differentiation. *J Exp Med.* 2020; 217(1): e20190832. 10.1084/jem.20190832 31636135 PMC7037247

[103] Aliahmad P Seksenyan A Kaye J: The many roles of TOX in the immune system. *Curr Opin Immunol.* 2012; 24(2): 173–7. 10.1016/j.coi.2011.12.001 22209117 PMC3319641

[104] Seo H Chen J González-Avalos E: TOX and TOX2 transcription factors cooperate with NR4A transcription factors to impose CD8^+^ T cell exhaustion. *Proc Natl Acad Sci U S A.* 2019; 116(25): 12410–5. 10.1073/pnas.1905675116 31152140 PMC6589758

[105] Khan O Giles JR McDonald S: TOX transcriptionally and epigenetically programs CD8^+^ T cell exhaustion. *Nature.* 2019; 571(7764): 211–8. 10.1038/s41586-019-1325-x 31207603 PMC6713202

[106] Scott AC Dündar F Zumbo P: TOX is a critical regulator of tumour-specific T cell differentiation. *Nature.* 2019; 571(7764): 270–4. 10.1038/s41586-019-1324-y 31207604 PMC7698992

[107] Alfei F Kanev K Hofmann M: TOX reinforces the phenotype and longevity of exhausted T cells in chronic viral infection. *Nature.* 2019; 571(7764): 265–9. 10.1038/s41586-019-1326-9 31207605

[108] Yao C Sun Hw Lacey NE: Single-cell RNA-seq reveals TOX as a key regulator of CD8^+^ T cell persistence in chronic infection. *Nat Immunol.* 2019; 20(7): 890–901. 10.1038/s41590-019-0403-4 31209400 PMC6588409

[109] Xu W Zhao X Wang X: The Transcription Factor Tox2 Drives T Follicular Helper Cell Development via Regulating Chromatin Accessibility. *Immunity.* 2019; 51(5): 826–839.e5. 10.1016/j.immuni.2019.10.006 31732165

[110] He X He X Dave VP: The zinc finger transcription factor *Th-POK* regulates CD4 versus CD8 T-cell lineage commitment. *Nature.* 2005; 433(7028): 826–33. 10.1038/nature03338 15729333

[111] Sun G Liu X Mercado P: The zinc finger protein cKrox directs CD4 lineage differentiation during intrathymic T cell positive selection. *Nat Immunol.* 2005; 6(4): 373–81. 10.1038/ni1183 15750595

[112] Vacchio MS Ciucci T Gao Y: A Thpok-Directed Transcriptional Circuitry Promotes Bcl6 and Maf Expression to Orchestrate T Follicular Helper Differentiation. *Immunity.* 2019; 51(3): 465–478.e6. 10.1016/j.immuni.2019.06.023 31422869 PMC6904114

[113] Chan I: The role of extracellular matrix protein 1 in human skin. *Clin Exp Dermatol.* 2004; 29(1): 52–6. 10.1111/j.1365-2230.2004.01440.x 14723723

[114] Li Z Zhang Y Liu Z: ECM1 controls T_H_2 cell egress from lymph nodes through re-expression of S1P(1). *Nat Immunol.* 2011; 12(2): 178–85. 10.1038/ni.1983 21217760

[115] Su P Chen S Zheng YH: Novel Function of Extracellular Matrix Protein 1 in Suppressing Th17 Cell Development in Experimental Autoimmune Encephalomyelitis. *J Immunol.* 2016; 197(4): 1054–64. 10.4049/jimmunol.1502457 27316685 PMC4975973

[116] He L Gu W Wang M: Extracellular matrix protein 1 promotes follicular helper T cell differentiation and antibody production. *Proc Natl Acad Sci U S A.* 2018; 115(34): 8621–6. 10.1073/pnas.1801196115 30087185 PMC6112729

[117] Navarro MN Feijoo-Carnero C Arandilla AG: Protein kinase D2 is a digital amplifier of T cell receptor-stimulated diacylglycerol signaling in naïve CD8^+^ T cells. *Sci Signal.* 2014; 7(348): ra99. 10.1126/scisignal.2005477 25336615 PMC4768351

[118] Misawa T SoRelle JA Choi JH: Mutual inhibition between Prkd2 and Bcl6 controls T follicular helper cell differentiation. *Sci Immunol.* 2020; 5(43): eaaz0085. 10.1126/sciimmunol.aaz0085 31980486 PMC7278039

[119] Crotty S Kersh EN Cannons J: SAP is required for generating long-term humoral immunity. *Nature.* 2003; 421(6920): 282–7. 10.1038/nature01318 12529646

[120] Qi H Cannons JL Klauschen F: SAP-controlled T-B cell interactions underlie germinal centre formation. *Nature.* 2008; 455(7214): 764–9. 10.1038/nature07345 18843362 PMC2652134

[121] Preite S Cannons JL Radtke AJ: Hyperactivated PI3Kδ promotes self and commensal reactivity at the expense of optimal humoral immunity. *Nat Immunol.* 2018; 19(9): 986–1000. 10.1038/s41590-018-0182-3 30127432 PMC6140795

[122] Zeng H Cohen S Guy C: mTORC1 and mTORC2 Kinase Signaling and Glucose Metabolism Drive Follicular Helper T Cell Differentiation. *Immunity.* 2016; 45(3): 540–54. 10.1016/j.immuni.2016.08.017 27637146 PMC5050556

[123] Shi J Hou S Fang Q: PD-1 Controls Follicular T Helper Cell Positioning and Function. *Immunity.* 2018; 49(2): 264–274.e4. 10.1016/j.immuni.2018.06.012 30076099 PMC6104813

[124] Lu P Shih C Qi H: Ephrin B1-mediated repulsion and signaling control germinal center T cell territoriality and function. *Science.* 2017; 356(6339): eaai9264. 10.1126/science.aai9264 28408722

[125] Yan H Wu L Shih C: Plexin B2 and Semaphorin 4C Guide T Cell Recruitment and Function in the Germinal Center. *Cell Rep.* 2017; 19(5): 995–1007. 10.1016/j.celrep.2017.04.022 28467912

[126] Schrock DC Leddon SA Hughson A: Pivotal role for α_V_ integrins in sustained Tfh support of the germinal center response for long-lived plasma cell generation. *Proc Natl Acad Sci U S A.* 2019; 116(10): 4462–70. 10.1073/pnas.1809329116 30770452 PMC6410787

[127] Weisel FJ Zuccarino-Catania GV Chikina M: A Temporal Switch in the Germinal Center Determines Differential Output of Memory B and Plasma Cells. *Immunity.* 2016; 44(1): 116–30. 10.1016/j.immuni.2015.12.004 26795247 PMC4724390

[128] MacIver NJ Michalek RD Rathmell JC: Metabolic regulation of T lymphocytes. *Annu Rev Immunol.* 2013; 31: 259–83. 10.1146/annurev-immunol-032712-095956 23298210 PMC3606674

[129] Buck MD O'Sullivan D Pearce EL: T cell metabolism drives immunity. *J Exp Med.* 2015; 212(9): 1345–60. 10.1084/jem.20151159 26261266 PMC4548052

[130] Laplante M Sabatini DM: mTOR signaling in growth control and disease. *Cell.* 2012; 149(2): 274–93. 10.1016/j.cell.2012.03.017 22500797 PMC3331679

[131] Chi H: Regulation and function of mTOR signalling in T cell fate decisions. *Nat Rev Immunol.* 2012; 12(5): 325–38. 10.1038/nri3198 22517423 PMC3417069

[132] Ray JP Staron MM Shyer JA: The Interleukin-2-mTORc1 Kinase Axis Defines the Signaling, Differentiation, and Metabolism of T Helper 1 and Follicular B Helper T Cells. *Immunity.* 2015; 43(4): 690–702. 10.1016/j.immuni.2015.08.017 26410627 PMC4618086

[133] Hao Y Wang Y Liu X: The Kinase Complex mTOR Complex 2 Promotes the Follicular Migration and Functional Maturation of Differentiated Follicular Helper CD4^+^ T Cells During Viral Infection. *Front Immunol.* 2018; 9: 1127. 10.3389/fimmu.2018.01127 29875775 PMC5974104

[134] Kitano M Moriyama S Ando Y: Bcl6 protein expression shapes pre-germinal center B cell dynamics and follicular helper T cell heterogeneity. *Immunity.* 2011; 34(6): 961–72. 10.1016/j.immuni.2011.03.025 21636294

[135] Lüthje K Kallies A Shimohakamada Y: The development and fate of follicular helper T cells defined by an IL-21 reporter mouse. *Nat Immunol.* 2012; 13(5): 491–8. 10.1038/ni.2261 22466669

[136] Shulman Z Gitlin AD Targ S: T follicular helper cell dynamics in germinal centers. *Science.* 2013; 341(6146): 673–7. 10.1126/science.1241680 23887872 PMC3941467

[137] Oestreich KJ Read KA Gilbertson SE: Bcl-6 directly represses the gene program of the glycolysis pathway. *Nat Immunol.* 2014; 15(10): 957–64. 10.1038/ni.2985 25194422 PMC4226759

[138] Yi W Gupta S Ricker E: The mTORC1-4E-BP-eIF4E axis controls de novo Bcl6 protein synthesis in T cells and systemic autoimmunity. *Nat Commun.* 2017; 8(1): 254. 10.1038/s41467-017-00348-3 28811467 PMC5557982

[139] Cho SH Raybuck AL Stengel K: Germinal centre hypoxia and regulation of antibody qualities by a hypoxia response system. *Nature.* 2016; 537(7619): 234–8. 10.1038/nature19334 27501247 PMC5161594

[140] Cho SH Raybuck AL Blagih J: Hypoxia-inducible factors in CD4^+^ T cells promote metabolism, switch cytokine secretion, and T cell help in humoral immunity . *Proc Natl Acad Sci U S A.* 2019; 116(18): 8975–84. 10.1073/pnas.1811702116 30988188 PMC6500120

[141] Sitkovsky M Ohta A: Targeting the hypoxia-adenosinergic signaling pathway to improve the adoptive immunotherapy of cancer. *J Mol Med (Berl).* 2013; 91(2): 147–55. 10.1007/s00109-013-1001-9 23334369 PMC3576025

[142] Abbott RK Silva M Labuda J: The GS Protein-coupled A2a Adenosine Receptor Controls T Cell Help in the Germinal Center. *J Biol Chem.* 2017; 292(4): 1211–7. 10.1074/jbc.C116.764043 27974461 PMC5270467

[143] Alam MS Costales MG Cavanaugh C: Extracellular adenosine generation in the regulation of pro-inflammatory responses and pathogen colonization. *Biomolecules.* 2015; 5(2): 775–92. 10.3390/biom5020775 25950510 PMC4496696

[144] Proietti M Cornacchione V Rezzonico Jost T: ATP-gated ionotropic P2X7 receptor controls follicular T helper cell numbers in Peyer's patches to promote host-microbiota mutualism. *Immunity.* 2014; 41(5): 789–801. 10.1016/j.immuni.2014.10.010 25464855

[145] Victora GD Nussenzweig MC: Germinal centers. *Annu Rev Immunol.* 2012; 30: 429–57. 10.1146/annurev-immunol-020711-075032 22224772

[146] Victora GD Schwickert TA Fooksman DR: Germinal center dynamics revealed by multiphoton microscopy with a photoactivatable fluorescent reporter. *Cell.* 2010; 143(4): 592–605. 10.1016/j.cell.2010.10.032 21074050 PMC3035939

[147] Le TvL Kim TH Chaplin DD: Intraclonal competition inhibits the formation of high-affinity antibody-secreting cells. *J Immunol.* 2008; 181(9): 6027–37. 10.4049/jimmunol.181.9.6027 18941192 PMC2922957

[148] Awe O Hufford MM Wu H: PU.1 Expression in T Follicular Helper Cells Limits CD40L-Dependent Germinal Center B Cell Development. *J Immunol.* 2015; 195(8): 3705–15. 10.4049/jimmunol.1500780 26363052 PMC4592843

[149] Meli AP Fontés G Avery DT: The Integrin LFA-1 Controls T Follicular Helper Cell Generation and Maintenance. *Immunity.* 2016; 45(4): 831–46. 10.1016/j.immuni.2016.09.018 27760339 PMC5672956

[150] Cao Y Yang Q Deng H: Transcriptional factor ATF3 protects against colitis by regulating follicular helper T cells in Peyer's patches. *Proc Natl Acad Sci U S A.* 2019; 116(13): 6286–91. 10.1073/pnas.1818164116 30862736 PMC6442638

[151] Zotos D Coquet JM Zhang Y: IL-21 regulates germinal center B cell differentiation and proliferation through a B cell-intrinsic mechanism. *J Exp Med.* 2010; 207(2): 365–78. 10.1084/jem.20091777 20142430 PMC2822601

[152] Linterman MA Beaton L Di Yu: IL-21 acts directly on B cells to regulate Bcl-6 expression and germinal center responses. *J Exp Med.* 2010; 207(2): 353–63. 10.1084/jem.20091738 20142429 PMC2822609

[153] Reinhardt RL Liang HE Locksley RM: Cytokine-secreting follicular T cells shape the antibody repertoire. *Nat Immunol.* 2009; 10(4): 385–93. 10.1038/ni.1715 19252490 PMC2714053

[154] King IL Mohrs M: IL-4-producing CD4^+^ T cells in reactive lymph nodes during helminth infection are T follicular helper cells. *J Exp Med.* 2009; 206(5): 1001–7. 10.1084/jem.20090313 19380638 PMC2715031

[155] Glatman Zaretsky A Taylor JJ King IL: T follicular helper cells differentiate from Th2 cells in response to helminth antigens. *J Exp Med.* 2009; 206(5): 991–9. 10.1084/jem.20090313 19380637 PMC2715032

[156] Weinstein JS Herman EI Lainez B: TFH cells progressively differentiate to regulate the germinal center response. *Nat Immunol.* 2016; 17(10): 1197–1205. 10.1038/ni.3554 27573866 PMC5030190

[157] Gowthaman U Chen JS Zhang B: Identification of a T follicular helper cell subset that drives anaphylactic IgE. *Science.* 2019; 365(6456): eaaw6433. 10.1126/science.aaw6433 31371561 PMC6901029

[158] Yusuf I Kageyama R Monticelli L: Germinal center T follicular helper cell IL-4 production is dependent on signaling lymphocytic activation molecule receptor (CD150). *J Immunol.* 2010; 185(1): 190–202. 10.4049/jimmunol.0903505 20525889 PMC2913439

[159] Zheng WP Flavell RA: The Transcription Factor GATA-3 Is Necessary and Sufficient for Th2 Cytokine Gene Expression in CD4 T Cells. *Cell.* 1997; 89(4): 587–96. 10.1016/s0092-8674(00)80240-8 9160750

[160] Clement RL Daccache J Mohammed MT: Follicular regulatory T cells control humoral and allergic immunity by restraining early B cell responses. *Nat Immunol.* 2019; 20(10): 1360–71. 10.1038/s41590-019-0472-4 31477921 PMC6754271

[161] Liang H Tang J Liu Z: ZIKV infection induces robust Th1-like Tfh cell and long-term protective antibody responses in immunocompetent mice. *Nat Commun.* 2019; 10(1): 3859. 10.1038/s41467-019-11754-0 31455769 PMC6712032

[162] Weinstein JS Laidlaw BJ Lu Y: STAT4 and T-bet control follicular helper T cell development in viral infections. *J Exp Med.* 2018; 215(1): 337–55. 10.1084/jem.20170457 29212666 PMC5748849

[163] Sheikh AA Cooper L Feng M: Context-Dependent Role for T-bet in T Follicular Helper Differentiation and Germinal Center Function following Viral Infection. *Cell Rep.* 2019; 28(7): 1758–1772.e4. 10.1016/j.celrep.2019.07.034 31412245 PMC6711398

[164] Wang P Wang Y Xie L: The Transcription Factor T-Bet Is Required for Optimal Type I Follicular Helper T Cell Maintenance During Acute Viral Infection. *Front Immunol.* 2019; 10: 606. 10.3389/fimmu.2019.00606 30984183 PMC6449430

[165] Elsner RA Shlomchik MJ: IL-12 Blocks Tfh Cell Differentiation during *Salmonella* Infection, thereby Contributing to Germinal Center Suppression. *Cell Rep.* 2019; 29(9): 2796–2809.e5. 10.1016/j.celrep.2019.10.069 31775046 PMC6908568

[166] Fang D Cui K Mao K: Transient T-bet expression functionally specifies a distinct T follicular helper subset. *J Exp Med.* 2018; 215(11): 2705–14. 10.1084/jem.20180927 30232200 PMC6219743

[167] Papillion A Powell MD Chisolm DA: Inhibition of IL-2 responsiveness by IL-6 is required for the generation of GC-T*FH* cells. *Sci Immunol.* 2019; 4(39): eaaw7636. 10.1126/sciimmunol.aaw7636 31519812 PMC6820141

[168] Fazilleau N Eisenbraun MD Malherbe L: Lymphoid reservoirs of antigen-specific memory T helper cells. *Nat Immunol.* 2007; 8(7): 753–61. 10.1038/ni1472 17529982

[169] Hale JS Youngblood B Latner DR: Distinct memory CD4^+^ T cells with commitment to T follicular helper- and T helper 1-cell lineages are generated after acute viral infection. *Immunity.* 2013; 38(4): 805–17. 10.1016/j.immuni.2013.02.020 23583644 PMC3741679

[170] Asrir A Aloulou M Gador M: Interconnected subsets of memory follicular helper T cells have different effector functions. *Nat Commun.* 2017; 8(1): 847. 10.1038/s41467-017-00843-7 29018187 PMC5635037

[171] Ciucci T Vacchio MS Gao Y: The Emergence and Functional Fitness of Memory CD4^+^ T Cells Require the Transcription Factor Thpok. *Immunity.* 2019; 50(1): 91–105.e4. 10.1016/j.immuni.2018.12.019 30638736 PMC6503975

[172] Künzli M Schreiner D Pereboom TC: Long-lived T follicular helper cells retain plasticity and help sustain humoral immunity. *Sci Immunol.* 2020; 5(45): eaay5552. 10.1126/sciimmunol.aay5552 32144185

[173] Cirelli KM Carnathan DG Nogal B: Slow Delivery Immunization Enhances HIV Neutralizing Antibody and Germinal Center Responses via Modulation of Immunodominance. *Cell.* 2019; 177(5): 1153–1171.e28. 10.1016/j.cell.2019.04.012 31080066 PMC6619430

